# Author Correction: Upregulation of the AMPK-FOXO1-PDK4 pathway is a primary mechanism of pyruvate dehydrogenase activity reduction in tafazzin-deficient cells

**DOI:** 10.1038/s41598-024-65477-4

**Published:** 2024-06-25

**Authors:** Zhuqing Liang, Tyler Ralph-Epps, Michael W. Schmidtke, Pablo Lazcano, Simone W. Denis, Mária Balážová, Nevton Teixeira da Rosa, Mohamed Chakkour, Sanaa Hazime, Mindong Ren, Michael Schlame, Riekelt H. Houtkooper, Miriam L. Greenberg

**Affiliations:** 1 Department of Biological Sciences, Wayne State University, Detroit, MI USA; 2 Laboratory Genetic Metabolic Diseases, Amsterdam UMC, University of Amsterdam, Amsterdam, The Netherlands; 3 Amsterdam Gastroenterology Endocrinology and Metabolism Institute, Amsterdam, The Netherlands; 4 Amsterdam Cardiovascular Sciences Institute, Amsterdam, The Netherlands; 5 Emma Center for Personalized Medicine, Amsterdam UMC, Amsterdam, The Netherlands; 6 Department of Membrane Biochemistry, Institute of Animal Biochemistry and Genetics, Centre of Biosciences, Slovak Academy of Sciences, 84005 Bratislava, Slovakia; 7 Department of Anesthesiology, Perioperative Care, and Pain Medicine, Grossman School of Medicine, New York University, New York, NY USA

Correction to: *Scientific Reports* 10.1038/s41598-024-62262-1, published online 20 May 2024

The original version of this article contained an error in the name of the author Nevton Teixeira da Rosa Jr., wherein the printed order of 'Teixeira' and 'da Rosa' was reversed and the name suffix ‘Jr.’ was incorrectly stated as ‘J.’

The original Article has been corrected.

