# Supplementary figures and images for: Upregulation of the AMPK-FOXO1-PDK4 pathway is a primary mechanism of pyruvate dehydrogenase activity reduction in tafazzin-deficient cells
Source: Sci Rep. 2024 May 20;14:11497. doi: 10.1038/s41598-024-62262-1 (PMC11106297; doi:10.1038/s41598-024-62262-1)

FigS1

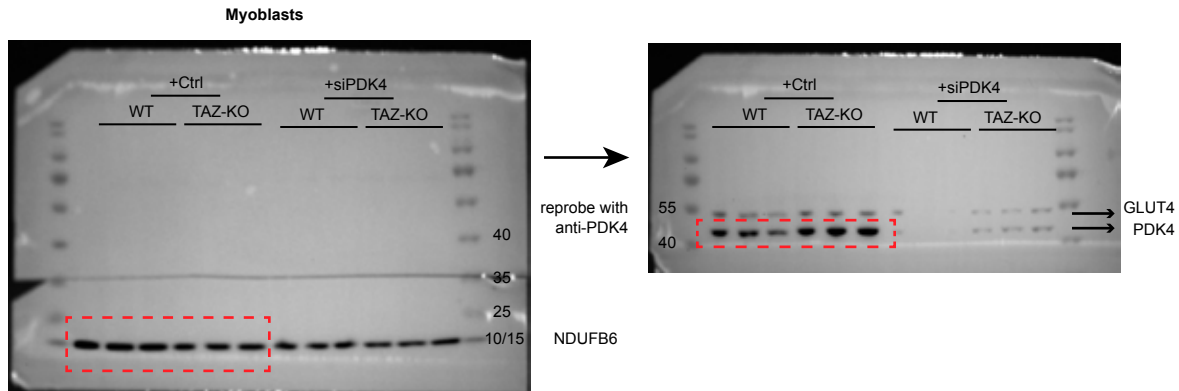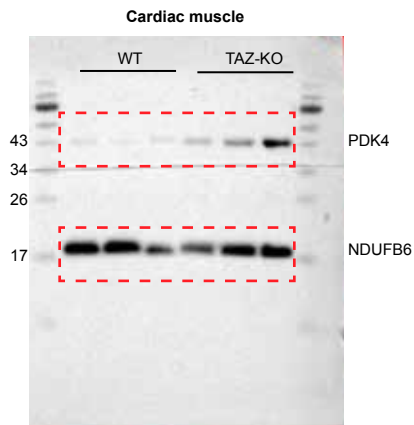

Supplement: Supplementary file 1 — Supplementary Figure S1. [file 41598_2024_62262_MOESM1_ESM.pdf]

FigS2

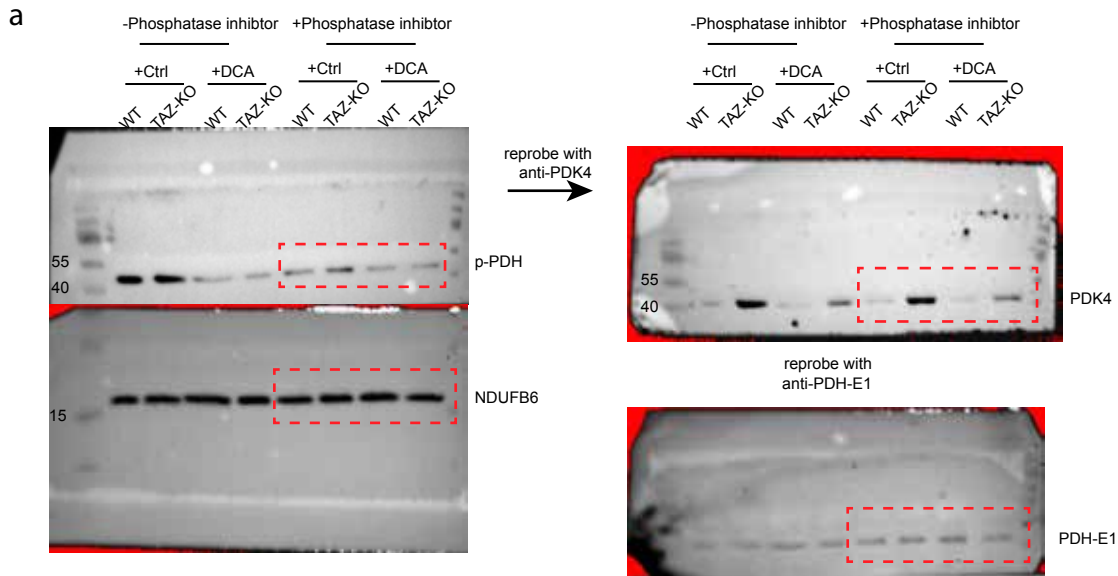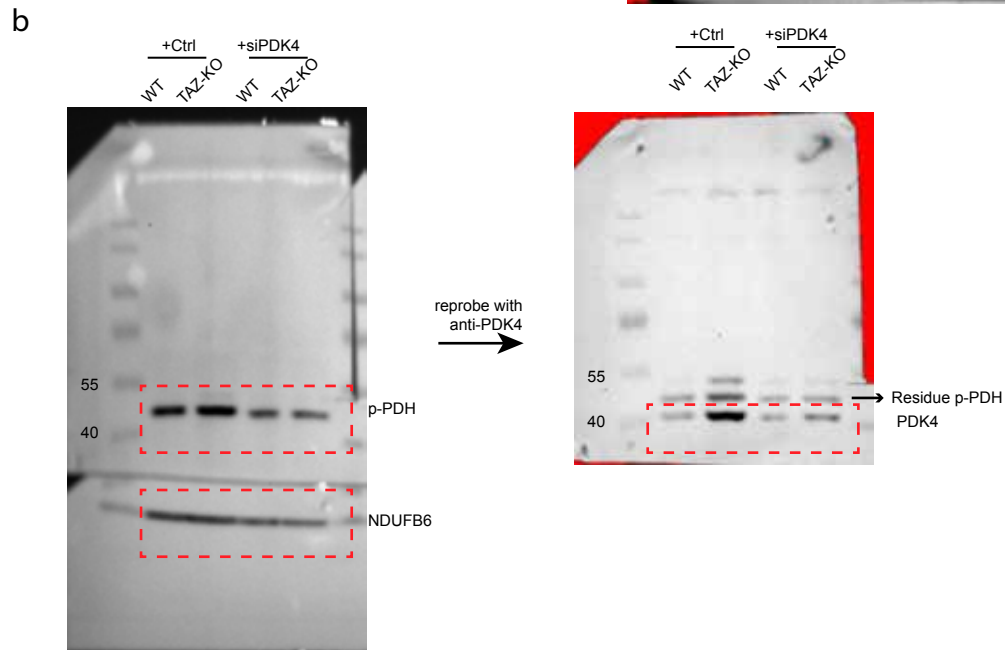

Supplement: Supplementary file 2 — Supplementary Figure S2. [file 41598_2024_62262_MOESM2_ESM.pdf]

FigS3

a

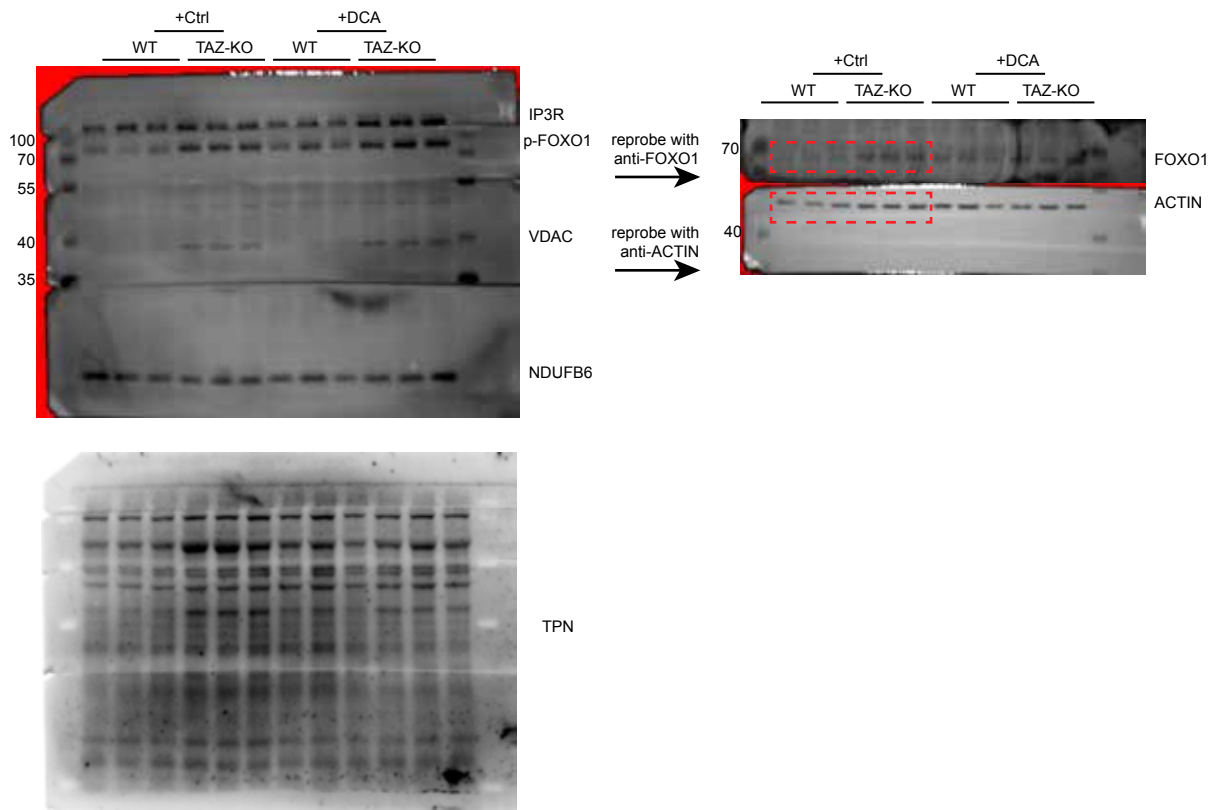

b

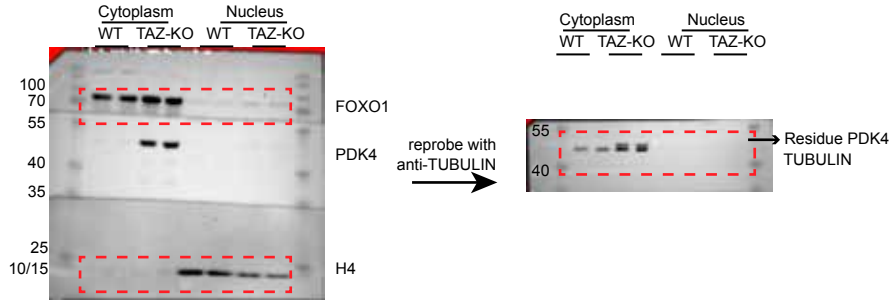

Supplement: Supplementary file 3 — Supplementary Figure S3. [file 41598_2024_62262_MOESM3_ESM.pdf]

Fig S4

**Cardiac muscle**

WT

TAZ-KO

WT

TAZ-KO

70

FOXO1

70

TPN

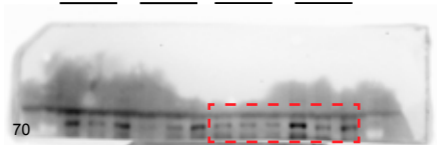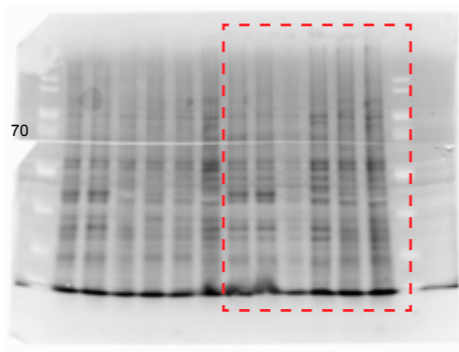

Supplement: Supplementary file 4 — Supplementary Figure S4. [file 41598_2024_62262_MOESM4_ESM.pdf]

Fig S5

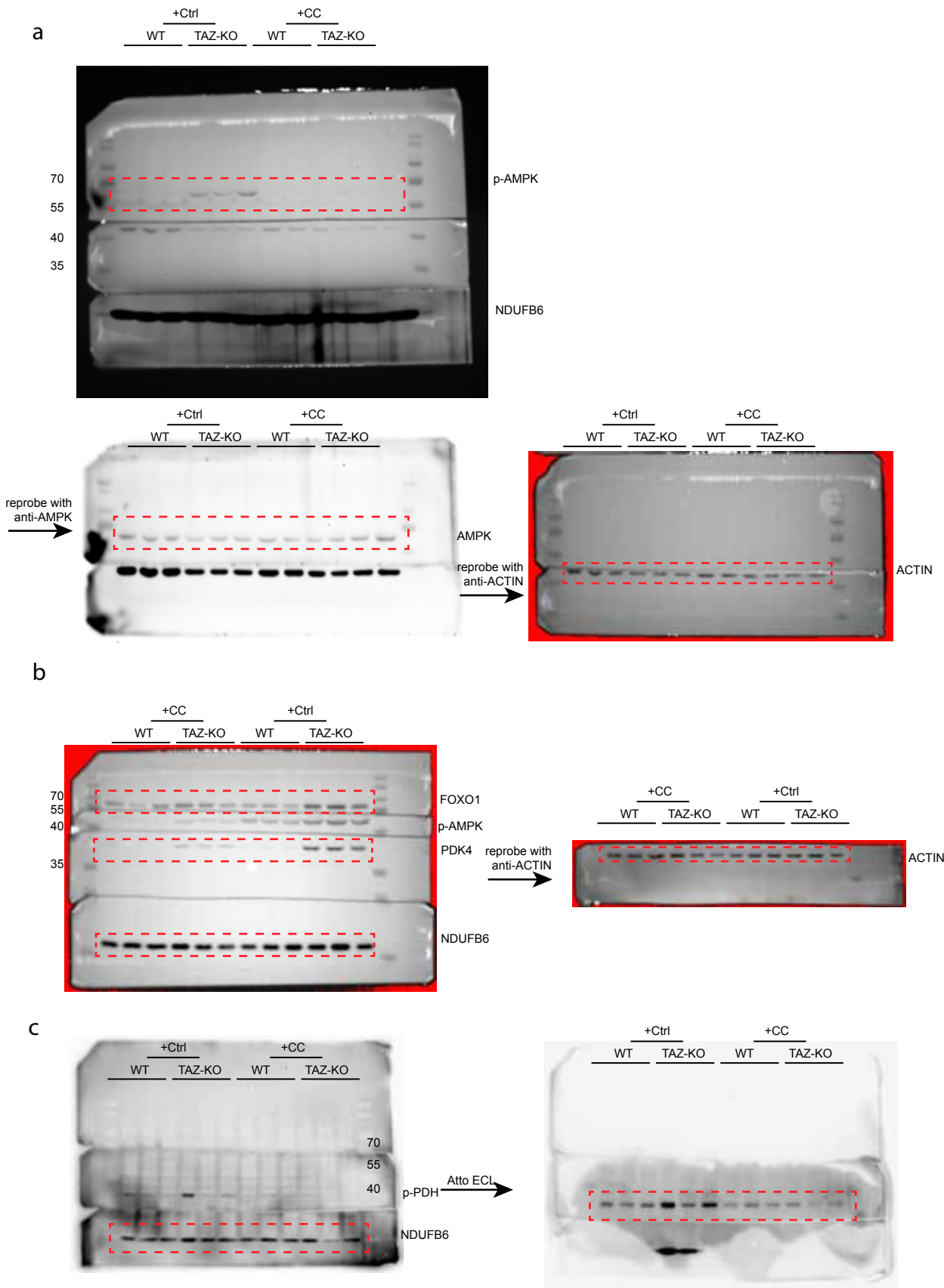

Supplement: Supplementary file 5 — Supplementary Figure S5. [file 41598_2024_62262_MOESM5_ESM.pdf]

Fig S6

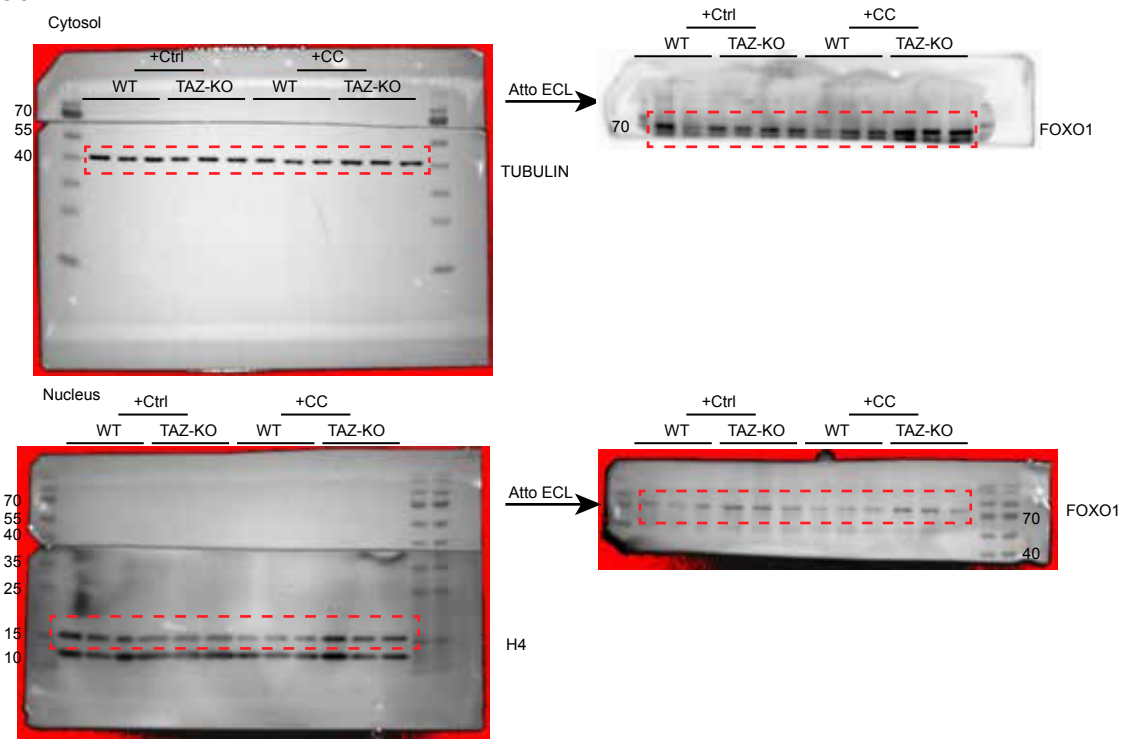

Supplement: Supplementary file 6 — Supplementary Figure S6. [file 41598_2024_62262_MOESM6_ESM.pdf]

Fig S7

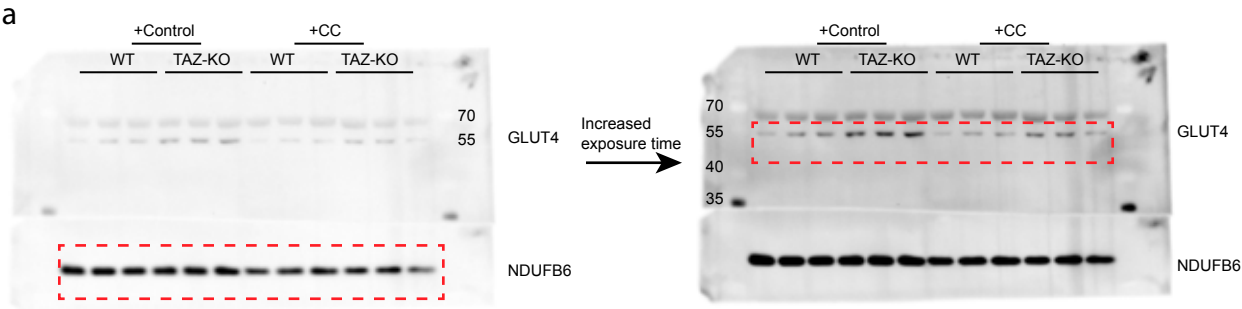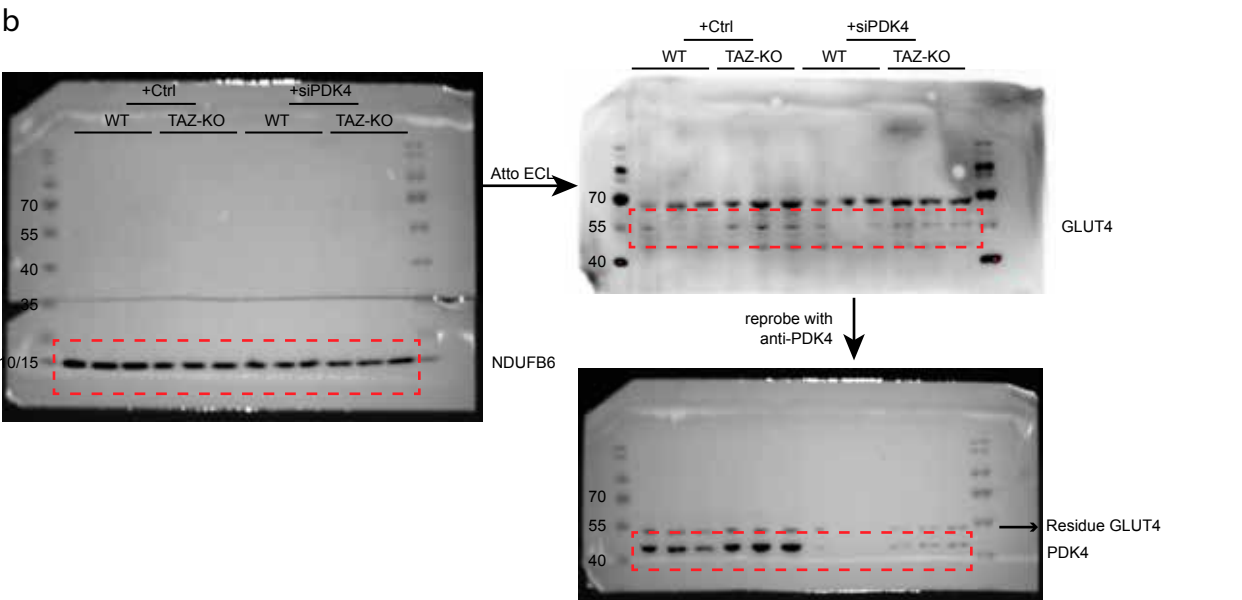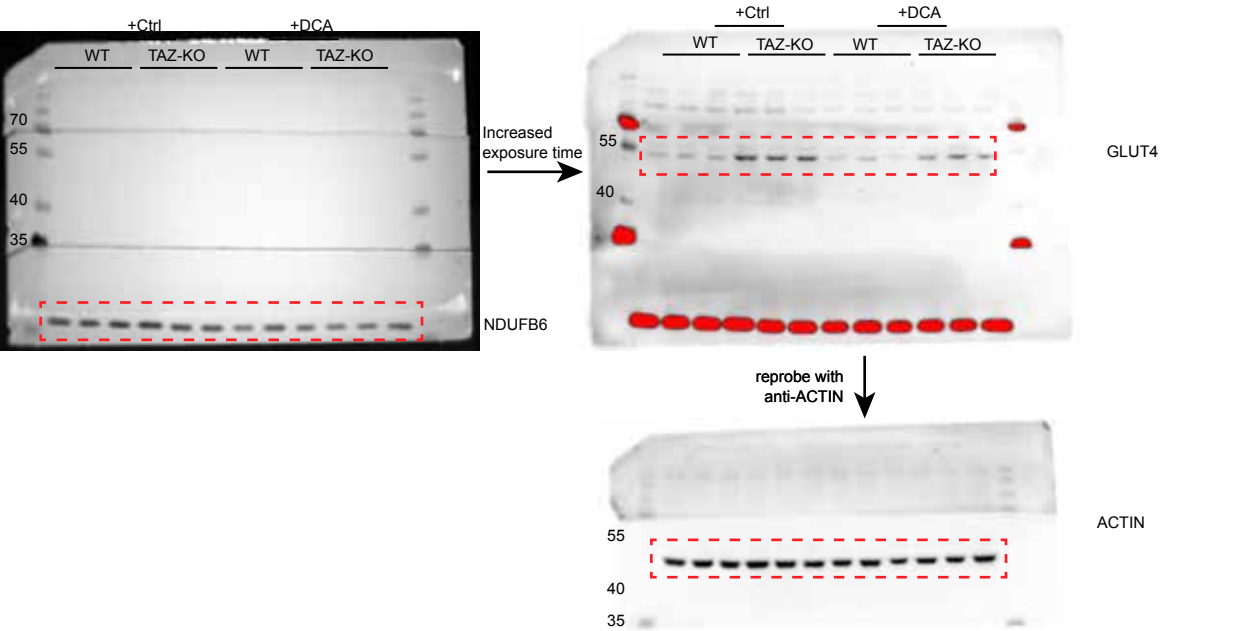

Supplement: Supplementary file 7 — Supplementary Figure S7. [file 41598_2024_62262_MOESM7_ESM.pdf]

Fig S8

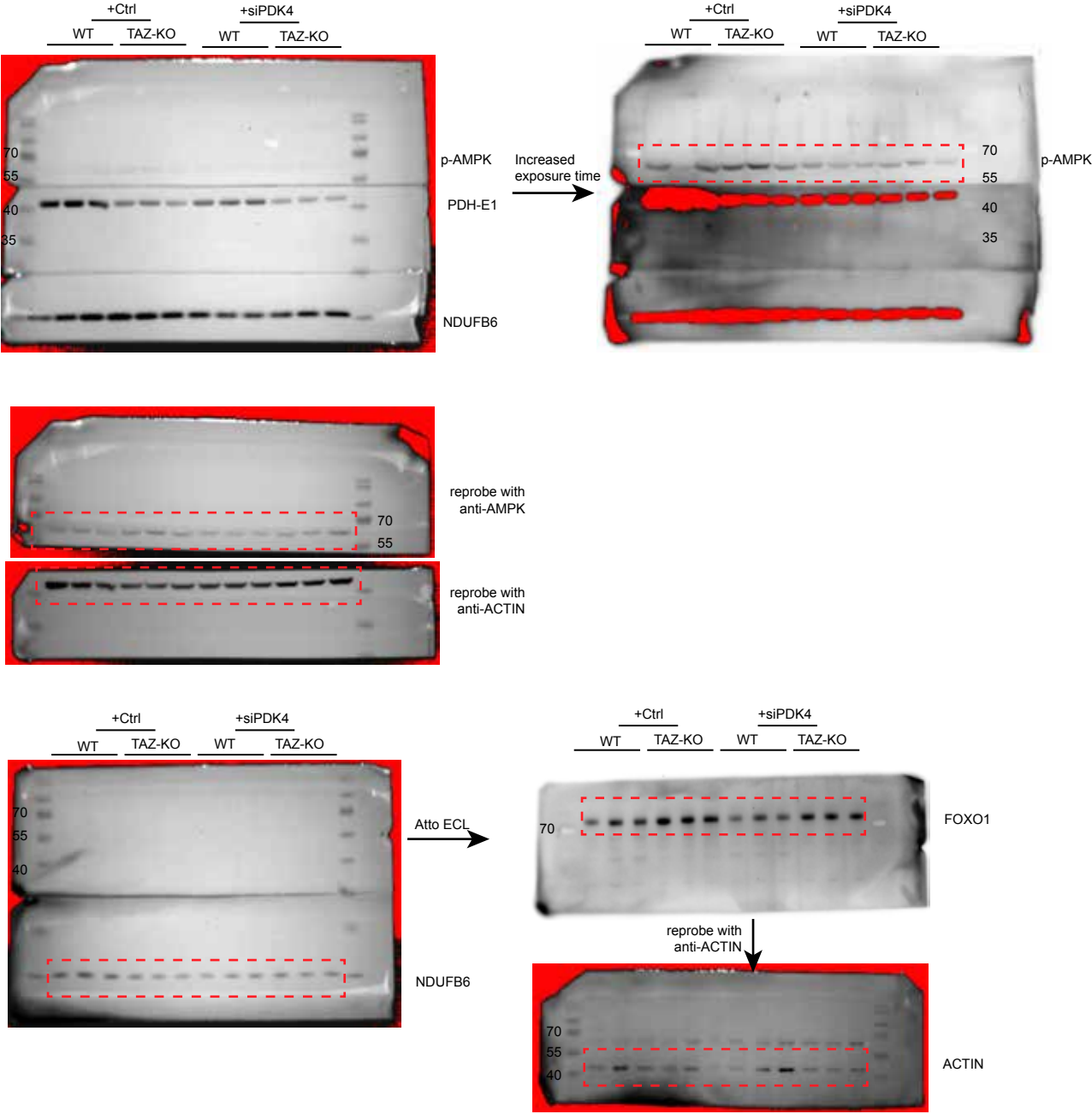

Supplement: Supplementary file 8 — Supplementary Figure S8. [file 41598_2024_62262_MOESM8_ESM.pdf]
