# Supplementary material for: Upregulation of the AMPK-FOXO1-PDK4 pathway is a primary mechanism of pyruvate dehydrogenase activity reduction in tafazzin-deficient cells
Source: Sci Rep. 2024 May 20;14:11497. doi: 10.1038/s41598-024-62262-1 (PMC11106297; doi:10.1038/s41598-024-62262-1)
Supplement: Supplementary file 9 — Supplementary Legends. [file 41598_2024_62262_MOESM9_ESM.docx]

**Supplementary Figure S1 Unedited images of Figure 1C**

Western blot (WB) analysis of whole-cell lysates extracted from WT and TAZ-KO myoblasts and mouse muscle tissue. The membrane was first probed with anti-GLUT4 (66846; Proteintech; anti-rabbit), and after mild stripping, re-probed with anti-PDK4(ab214938; Abcam; anti-rabbit). Red boxes indicate the cropped regions of the membranes presented in Fig. 1C.

**Supplementary Figure S2 Unedited images of Figure 2A and 2B**

WT and TAZ-KO myoblasts were treated with either 5 mM Dichloroacetate (DCA) for 16 h (a) or PDK4-targeted siRNA for 24 h (b). Phosphorylation of PDH on Ser^293^ (p-PDH) was assessed via WB probing with indicated antibody (anti-p-PDH, AP1062; Millipore; anti-rabbit), and after mild stripping, re-probed with anti-PDH-E1 (sc377092; Santa Cruz; anti-mouse) and anti-PDK4 (ab214938; Abcam; anti-rabbit). Red boxes indicate the cropped regions of the membranes presented in Fig. 2A and B.

**Supplementary Figure S3 Unedited images of Figure 3A and 3C**

(a) Whole-cell lysates were extracted from WT and TAZ-KO myoblasts, the protein levels of FOXO1 were measured using WB analysis. The membrane was initially probed with anti-p-FOXO1 (9461; Cell Signaling; anti-rabbit) and anti-VDAC (sc390996; Santa Cruz; anti-mouse), then after mild stripping, re-probed with anti-FOXO1 (14952; Cell Signaling; anti-mouse) and ACTIN (sc47778; Santa Cruz; anti-mouse). TPN stands for total protein normalization stain. (b) Nuclear protein fractionation was performed, followed by WB analysis using tubulin and histone 4 (H4) as internal controls for cytoplasmic and nuclear fractions, respectively. The membrane was first probed with anti-PDK4 (ab214938; Abcam; anti-rabbit), and after mild stripping, re-probed with anti-TUBULIN (ab184970; Abcam; anti-mouse). Red boxes indicate the cropped regions of the membranes presented in Fig. 3A and C.

**Supplementary Figure S4 Unedited images of Figure 4B**

Whole-cell lysates were extracted from WT and TAZ-KO mouse cardiac tissues, and FOXO1 protein levels were measured using WB analysis. A total protein stain was used for WB analysis normalization (TPN). Red boxes indicate the cropped regions of the membranes presented in Fig. 4B.

**Supplementary Figure S5 Unedited images of Figure 5A, C and D**

(a) Phosphorylation of AMPK residue Thr^172^ (p-AMPK) was assayed in WT and TAZ-KO myoblasts treated with the AMPK inhibitor compound C (CC) or vehicle. The membrane was first probed with anti-p-AMPK (2535S; Cell Signaling; anti-rabbit), and after mild stripping, re-probed with anti-AMPK (2793S; Cell Signaling; anti-mouse) and anti-ACTIN (sc47778; Santa Cruz; anti-mouse). (b) Protein expression of FOXO1 and PDK4 in myoblasts was measured via WB analysis following treatment with CC (10 μM, 16 h) or vehicle. The membrane was first probed with anti-PDK4 (ab214938; Abcam; anti-rabbit), and after mild stripping, re-probed with anti-ACTIN (sc47778; Santa Cruz; anti-mouse). (c) In myoblasts, p-PDH was measured by WB analysis following treatment with CC (10 μM, 16 h) or vehicle. Atto ECL used was SuperSignal™ West Atto Ultimate Sensitivity Substrate (A38554). Red boxes indicate the cropped regions of the membranes presented in Fig. 5A, C and D.

**Supplementary Figure S6 Unedited images of Figure 6B**

FOXO1 protein levels were assayed in nuclear and cytoplasmic cellular fractions following treatment with CC (5 μM, 16 h) or vehicle. Tubulin and histone H3 were used as internal controls for cytoplasmic and nuclear fractions. Atto ECL used was SuperSignal™ West Atto Ultimate Sensitivity Substrate (A38554). Red boxes indicate the cropped regions of the membranes presented in Fig. 6B.

**Supplementary Figure S7 Unedited images of Figure 7C and D**

GLUT4 protein expression was measured by WB in myoblasts treated with either 10 μM CC (a) or 5 mM DCA for 16 h or PDK4-targeted siRNA for 24 h (b). The membrane was first probed with anti-GLUT4 (66846; Proteintech; anti-rabbit), and after mild stripping, re-probed with anti-PDK4 (ab214938; Abcam; anti-rabbit) and anti-ACTIN (sc47778; Santa Cruz; anti-mouse). Atto ECL used was SuperSignal™ West Atto Ultimate Sensitivity Substrate (A38554). Red boxes indicate the cropped regions of the membranes presented in Fig. 7C and D.

**Supplementary Figure S8 Unedited images of Figure 8**

Phosphorylation of AMPK residue Thr^172^ (p-AMPK) and FOXO1 protein levels were assayed in WT and TAZ-KO myoblasts treated with PDK4-targeted siRNA for 24 h. The membrane was first probed with anti-p-AMPK (2535S; Cell Signaling; anti-rabbit), anti-PDH-E1 (sc377092; Santa Cruz; anti-mouse), and anti-FOXO1 (14952; Cell Signaling; anti-mouse), and after mild stripping, re-probed with anti-AMPK (2793S; Cell Signaling; anti-mouse) and anti-ACTIN (sc47778; Santa Cruz; anti-mouse). Atto ECL used was SuperSignal™ West Atto Ultimate Sensitivity Substrate (A38554). Red boxes indicate the cropped regions of the membranes presented in Fig. 8.
